# Imaging orofacial pain in mice

**DOI:** 10.1186/1744-8069-10-S1-O2

**Published:** 2014-12-15

**Authors:** Yu Shin Kim, Yuxia Chu, Liang Han, Kyougsook Park, Man Li, Zhe Li, Pamela Colleen LaVinka, Michael J Caterina, Ke Ren, Ronald Dubner, Feng Wei, Xinzhong Dong

**Affiliations:** 1 Department of Neuroscience, Center of Sensory Biology, The Johns Hopkins University School of Medicine, Baltimore, MD 21205, USA; 2 Department of Neural and Pain Sciences, Program in Neuroscience, Dental School, University of Maryland, Baltimore, MD 21201, USA; 3 Department of Anesthesiology, Tongji Hospital, Tongji Medical College, Huazhong University of Science and Technology, China; 4 Department of Biological Chemistry, The Johns Hopkins University School of Medicine, Baltimore, MD 21205, USA; 5 Howard Hughes Medical Institute, The Johns Hopkins University School of Medicine, Baltimore, MD 21205, USA

## 

Nociceptors in the dorsal root ganglia (DRG) and trigeminal ganglion (TG) play an essential role in initiating pain by detecting painful stimuli through their peripheral axons and sending signals to the spinal cord via their central axons [1]. Pathological conditions such as inflammation and nerve injury can sensitize nociceptors, causing heightened pain sensitivity and often leading to chronic pain conditions like TMJ disorders. Despite its importance in understanding the mechanism of nociceptor sensitization, monitoring neuronal activity of nociceptors in tissue explants or in live animals is still technically challenging due to the interference of the surrounding tissues. Recently, we have developed a novel approach to directly monitor neuronal activity and hyperactivity after injury and revealed the contribution of central terminal sensitization of primary nociceptive neurons to molecular mechanisms underlying the maintenance of trigeminal neuropathic pain. We generated *Pirt-GCaMP3* mice in which GCaMP3, a genetic-encoded Ca^2+^-sensitive indicator [2], is specifically expressed in >95% of all DRG and TG neurons under the Pirt promoter[3]. Because of the specific expression of the Ca^2+^ sensor (i.e., only in DRG and TG and not in skin cells or spinal cord neurons), we detected robust neuronal hyperexcitability in TG explants and TG’s axons in the skin explants and trigeminal brain-stem slices of animals with nerve injury compared with naïve or sham-treated mice. In addition, we are developing techniques to image DRG neuronal activity in live mice in response to various sensory stimuli applied to sensory peripheral receptive fields. The advantages of the functional imaging using *Pirt-GCaMP3* mice include simple tissue preparation and imaging procedures, intact sensory somatotopic organization, and simultaneously monitoring a large population of neurons and nerves. Previous and ongoing studies using this technique have revealed new mechanisms underlying chronic pain conditions including orofacial pain.

## Disclosures

Dr. Caterina is an inventor on a patent on the use of products related to TRPV1, which is licensed through UCSF and Merck, and may be entitled to royalties related to these products. He is on the Scientific Advisory Board for Hydra Biosciences, which develops products related to TRP channels. These conflicts are being managed by the Johns Hopkins Office on Policy Coordination.
